# Design and Performance Test of an Ocean Turbulent Kinetic Energy Dissipation Rate Measurement Probe

**DOI:** 10.3390/mi9060311

**Published:** 2018-06-20

**Authors:** Bian Tian, Huafeng Li, Hua Yang, Yulong Zhao, Pei Chen, Dalei Song

**Affiliations:** 1 State Key Laboratory for Manufacturing Systems Engineering, Xi’an Jiaotong University, Xi’an 710049, China; t.b12@mail.xjtu.edu.cn (B.T.); lihuafeng@stu.xjtu.edu.cn (H.L.); zhaoyulong@mail.xjtu.edu.cn (Y.Z.); 2 College of Information Science and Engineering, Ocean University of China, Qingdao 266100, China; 3 School of Construction Machinery, Chang’an University, Xi’an 710064, China; chdchenpei@chd.edu.cn; 4 College of Engineering, Ocean University of China, Qingdao 266100, China; songdalei@ouc.edu.cn

**Keywords:** turbulent kinetic energy dissipation rate, probe, microelectromechanical systems (MEMS) piezoresistive sensor chip, Taguchi method, marine environmental monitoring

## Abstract

Ocean turbulent kinetic energy dissipation rate is an essential parameter in marine environmental monitoring. Numerous probes have been designed to measure the turbulent kinetic energy dissipation rate in the past, and most of them utilize piezoelectric ceramics as the sensing element. In this paper, an ocean turbulent kinetic energy dissipation rate measurement probe utilizing a microelectromechanical systems (MEMS) piezoresistor as the sensing element has been designed and tested. The triangle cantilever beam and piezoresistive sensor chip are the core components of the designed probe. The triangle cantilever beam acts as a velocity-force signal transfer element, the piezoresistive sensor chip acts as a force-electrical signal transfer element, and the piezoresistive sensor chip is bonded on the triangle cantilever beam. One end of the triangle cantilever beam is a nylon sensing head which contacts with fluid directly, and the other end of it is a printed circuit board which processes the electrical signal. A finite element method has been used to study the effect of the cantilever beam on probe performance. The Taguchi optimization methodology is applied to optimize the structure parameters of the cantilever beam. An orthogonal array, signal-to-noise ratio, and analysis of variance are studied to analyze the effect of these parameters. Through the use of the designed probe, we can acquire the fluid flow velocity, and to obtain the ocean turbulent dissipation rate, an attached signal processing system has been designed. To verify the performance of the designed probe, tests in the laboratory and in the Bohai Sea are designed and implemented. The test results show that the designed probe has a measurement range of 10^−8^–10^−4^ W/kg and a sensitivity of 3.91 × 10^−4^ (Vms^2^)/kg. The power spectrum calculated from the measured velocities shows good agreement with the Nasmyth spectrum. The comparative analysis between the designed probe in this paper and the commonly used PNS probe has also been completed. The designed probe can be a strong candidate in marine environmental monitoring.

## 1. Introduction

The accurate measurement of the ocean turbulent dissipation rate is a key point in marine environmental monitoring. The turbulent kinetic energy dissipation rate represents the rate at which turbulent kinetic energy is converted into molecular thermal kinetic energy under the action of molecular viscosity, which can be used to build mathematical models to simulate the macroscopic motions of the ocean. Through the study of the turbulent kinetic energy dissipation rate, the ocean turbulent dissipation process can be constructed, which is of great importance to improve the physical model of the ocean and to study the law of mixing in the ocean. Ocean turbulence contributes significantly to the transport of momentum, heat, and mass in the ocean and has significant effects on the velocity, temperature characteristics, and distribution of dissolved and granular substances in the ocean.

Broadly used probes designed to measure the ocean turbulent kinetic energy dissipation rate utilize piezoelectric ceramics as the sensitive element. The first probe based on piezoelectric ceramics was designed by Ribner and Siddon [1,2], which was designed for atmospheric environmental monitoring. Osborn [3,4], at the University of British Columbia, studied the airfoil probe, which was a suitable and useful velocity sensor for oceanic turbulence. The probe had advantages over heated anemometry due to its rugged nature, lower susceptibility to fouling, and inherent linearity. However, improvements were also needed in the construction technique, so he improved the design of the former probe and tested it in Howe Sound near Vancouver, British Columbia. He concluded that the airfoil probe combined with a free-fall instrument housing was ideal for studying the vertical current shear in the ocean. The resolution and sensitivity were sufficient to provide estimates of the energy dissipation directly. This offers a classical approach to measuring the ocean turbulent dissipation rate. Wolk et al. [5] evaluated the performance of a free-falling microstructure profiler-TurboMAP, and the probe used on TurboMAP was almost exactly the same size and shape as the so-called Osborn probe. By assuming a universal form of the turbulence spectrum, turbulent kinetic energy dissipation rates below 5 × 10^−4^ W/kg can be estimated. Moum et al. [6], at Oregon State University, designed a shear sensor that utilizes piezobimorph ceramic as the sensing element which they named OSU, and they compared almost 1000 microstructure profiles obtained from different shear probes. Measurements of ocean microstructure were made in the turbulent Faroe Bank Channel overflow using a turbulence instrument equipped with SPM-38 turbulence shear probe [7]. The dissipation rate measurement results revealed that the lowest detection level was as low as 5 × 10^−11^ W/kg, which was comparable to the best available vertical microstructure profilers. Tianjin University in China designed a series of ocean turbulent dissipation rate measurement probes, such as TMR1, TJUA, and TJUB [8,9,10,11]. The TMR1 was the first ocean turbulence sensor probe they designed, and after that, they proposed the TJUA and the TJUB. Both the TMR1 and TJUA used piezoelectric ceramics as the sensitive material, and the TJUB used carbon fiber reinforced polymer plastic as the sensitive material, which lead to a high sensitivity of 2.52 × 10^−4^ (Vms^2^)/kg. The PNS series probes developed by Prandke at ISW Wassermesstechnik have been broadly used in marine environmental monitoring. PNS shear probes are airfoil-type microstructure velocity fluctuation sensors designed for a microstructure profiler. PNS-93 was specially designed for use in operational microstructure measuring systems. The use of a cantilever which transmits the lift force on the probe tip to the piezoceramic beam in the interior of the sensor was a huge step forward. The sensitivity of PNS-93 shear probe varied in a range from 1.5 × 10^−4^ to 2.1 × 10^−4^ (Vms^2^)/kg [12]. Then, a series of PNS probes were developed, such as PNS-98, PNS-03, and PNS-06. Cisewski et al. [13] investigated the mixing regime of the upper 180 m of a mesoscale eddy in the vicinity of the Antarctic Polar Front at 47° S and 21° E using the MSS profiler equipped with a microstructure shear sensor PNS-98. PNS-03 has an airfoil diameter and length of 3 mm and 4 mm, respectively, and PNS-06 has an airfoil diameter and length of 6 mm and 10 mm, respectively. PNS-03 and PNS-06 shear probes are available in a compact version and a version with thread M10. The sensitivities are in the order of 1 × 10^−4^ (Vms^2^)/kg for the PNS-03 and 4 × 10^−4^ (Vms^2^)/kg for the PNS-06 [14]. Now, the PNS series probes are the basic configurations of MSS-series microstructure turbulence profilers. Table 1 lists the detailed parameters of five series of probes and of the probe designed in this paper.

The aforementioned probes are almost based on the piezoelectric effect, and the probes based on piezoresistive effect are rarely mentioned. The signal processing system of piezoelectric-effect-based probes is complicated, but the signal processing system of piezoresistive-effect-based probes is simple. It is difficult for piezoelectric-effect-based probes to measure static signals, and the calibration also can be difficult. For the financial cost, the piezoresistive chip is suitable for mass production and micromation, but piezoelectric ceramics are sputtered and of high difficulty and high cost. In this paper, we designed an ocean turbulent kinetic energy dissipation rate measurement probe based on the piezoresistive effect. The triangle cantilever beam and piezoresistive sensor chip are the core components of the designed probe. An attached signal processing system has been designed and connected with a printed circuit board in the probe. Tests in the lab and in the ocean have been conducted separately. Test results reveal that the probe has good performance both in measurement range and sensitivity. The comparisons between the designed probe and the PNS probe have also been made.

## 2. Structure Design and Working Principle

**Figure 1:**
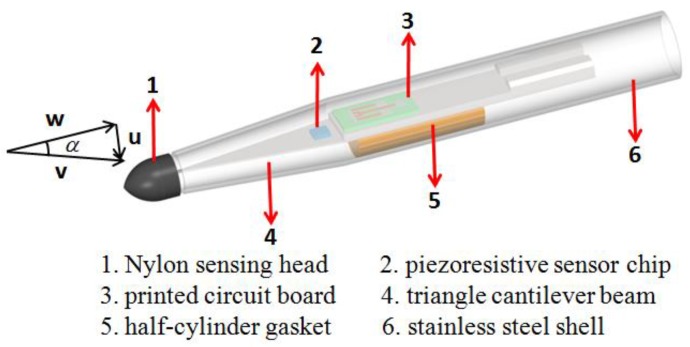
The structure of the designed ocean turbulent kinetic energy dissipation rate measurement probe.

The schematic diagram of the proposed ocean turbulent kinetic energy dissipation rate measurement probe is shown in Figure 1. The proposed probe consisted of a nylon sensing head, a piezoresistive sensor chip, a printed circuit board, a stainless-steel triangle cantilever beam, a half-cylinder gasket, and a stainless-steel shell. The nylon sensing head contacts with fluid flow mass directly and transforms the velocity signal to a force signal. The piezoresistive sensor chip was fabricated with an SOI (silicon-on-insulator) wafer, which consists of two layers of silicon and a silicon oxide in-between. The piezoresistive sensor chip was attached to the stainless-steel triangle cantilever beam, and a printed circuit board was also attached to it. The piezoresistive sensor chip was wire-bonded to the printed circuit board and further connected to a signal processing system. The piezoresistive sensor chip, printed circuit board, and stainless-steel triangle cantilever beam were packaged using a half-cylinder gasket and a stainless-steel shell, and the nylon sensing head was fixed on the free end of the stainless-steel triangle cantilever beam.

When there is a kind of fluid flow through the probe, according to the theory of fluid dynamics, assuming the fluid is nonviscous, the force produced by the fluid flow per unit length of the probe is [15]:

(1) $$f_{P}=\frac{1}{2}\rho v^{2}\frac{dA}{dx}\sin2\alpha$$

where *f_P_* is the force per unit length of probe, *ρ* is the density of the fluid, *v* is the velocity of the fluid flow, *dA/dx* is the rate of change in the cross-section area in the axial direction, and *α* is the angle of attack, as shown in Figure 1. In fact, the viscous effect also plays a role in terms of the kinetic energy dissipation, but the viscosity coefficient of water is very small and thus the viscous force produced by water is also very small [4]. The nonviscous fluid is considered as the flow fluid in this paper is to simplify the mathematical model. Then, the overall force on the probe is the integration from the bottom to the top of the probe:

(2) $$F=\int f_{P}dx=\frac{1}{2}\rho Av^{2}\sin2\alpha =\rho A(v\sin\alpha )(v\cos\alpha )=\rho Auw$$

where *u* is the cross velocity and *w* is the axial velocity.

When the fluid flows through the probe, the nylon sensing head produces deformation and thus the stainless-steel triangle cantilever beam produces deformation too. Then, the strain of the piezoresistive sensor chip changes and the variation of resistance of the piezoresistor is

(3) $$\frac{\Delta R}{R}=\pi F$$

where Δ*R* is the variation of resistance of the piezoresistor, *R* is the original value of the piezoresistor, and π is the piezoresistive coefficient of silicon. Then, the output voltage of piezoresistive sensor chip is

(4) $$U_{o}=U_{i}\frac{\Delta R}{R}=U_{i}\pi F=U_{i}\pi \rho Auw$$

where *U_o_* is the output voltage of the piezoresistive sensor chip and *U_i_* is the input voltage. From Equation (4), we know that the output voltage of the piezoresistive sensor chip is directly proportional to the cross velocity of fluid.

Defining *S* as the sensitivity of the designed probe, the sensitivity can be expressed as

(5) $$S=\frac{U_{o}}{u}=U_{i}\pi \rho Aw.$$

Equation (5) can be rewritten as

(6) $$u=\frac{U_{o}}{S}$$

then, the variation of cross velocity can be obtained as

(7) $$\frac{\partial u}{\partial t}=\frac{1}{S}\frac{\partial U_{o}}{\partial t}.$$

According to the Taylor frozen theory:

(8) $$\frac{\partial u}{\partial x}=\frac{1}{Sw}\frac{\partial U_{o}}{\partial t}.$$

**Figure 2:**
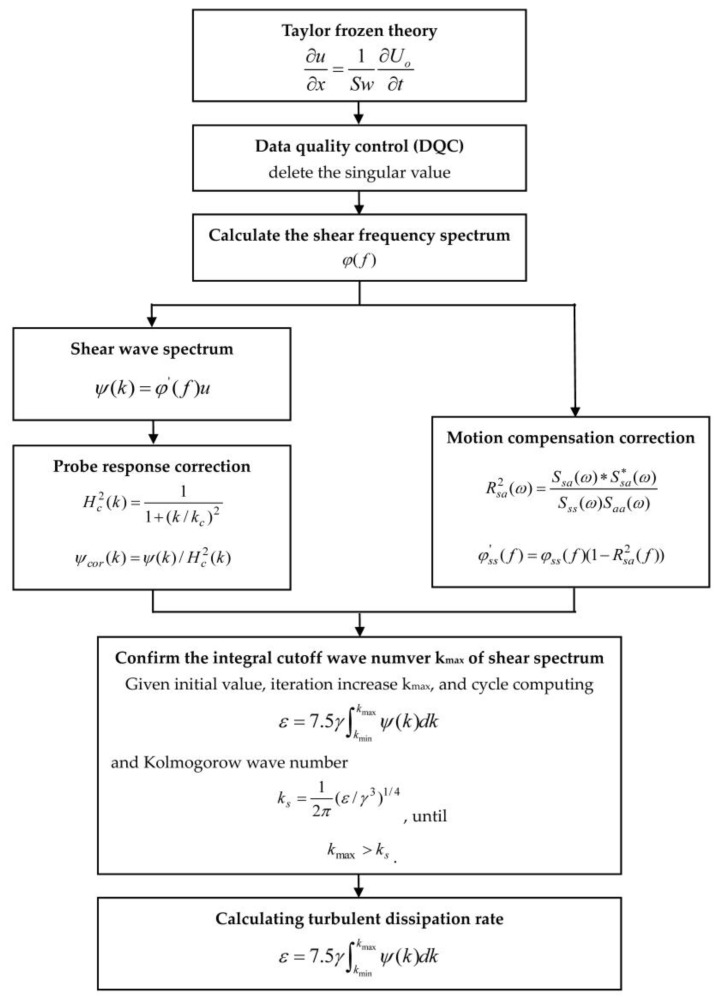
The flow chart of calculating turbulent kinetic energy dissipation rate.

Then, the turbulent kinetic energy dissipation rate can be calculated as shown in Figure 2. This is done by first deleting the singular value and calculating the shear frequency spectrum *ϕ*(*f*) in the frequency domain, followed by calculating the shear wavenumber spectrum *ψ*(*k*) in the wavenumber domain. Then, the probe response correction and motion compensation correction is completed, as shown in Figure 2. Finally, by confirming the integral cutoff wavenumber *k_max_* of the shear spectrum, the turbulent kinetic energy dissipation rate can be expressed as

(9) $$\varepsilon =7.5\gamma \int_{k_{\min}}^{k_{\max}}\psi (k)dk$$

where *ε* is the turbulent kinetic energy dissipation rate, *γ* is the kinematic viscosity coefficient, and *ψ*(*k*) is the power spectrum of the shear velocity.

**Figure 3:**
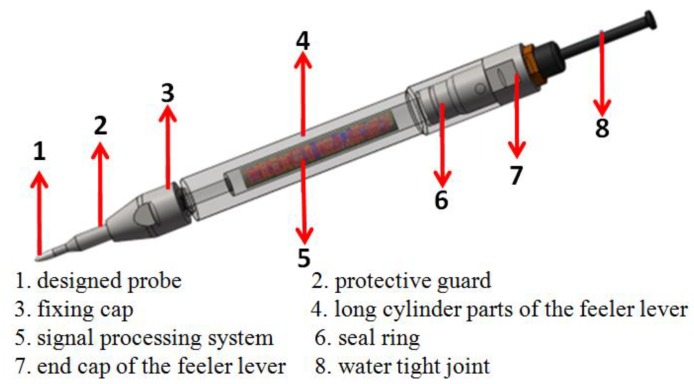
The overall diagram of the probe and signal processing system.

A signal processing system was designed and connected with the probe. The signal processing system main consisted of signal amplification, signal filtering, AD sample, reference source, single chip microcomputer, and power source. Further, some guard blocks were also designed, such as a protective guard, a fixing cap, a seal ring, and a water-tight joint. The overall diagram of the probe and the signal processing system is shown in Figure 3.

## 3. Finite Element Simulation and Optimal Design

According to the structure design and the working principle of the probe, the triangle cantilever beam has great influence on the performance of the probe. High sensitivity and high natural frequency are needed when the probe is working. Thus, the confirmation of the cantilever beam dimensions is an essential step in probe design. The Taguchi method was used to analyze and determine the dimensions of the cantilever beam. Taguchi uses a simple design of orthogonal array to study the entire parameter space with only a small number of experiments [16]. The greatest advantage of this method is that it saves effort in conducting experiments by reducing the experimental time, reducing the cost, and accelerating the pace at which significant factors are discovered [17]. In this paper, three parameters (height, thickness, and width of cantilever beam) at five levels were designed, and the fractional factorial design used was L_25_(5^3^) orthogonal array, as shown in Table 2.

We focused on the sensitivity of the probe, so the stress and deflection of the triangle cantilever beam when it was placed in the fluid flow was studied. Since we were concerned with the robustness of the probe, the natural frequency of the triangle cantilever beam had to be analyzed. The COMSOL Multiphysics (Version 5.3a, COMSOL Inc., Stockholm, Sweden) was used to simulate the results. In the simulation, water was used as the flow fluid. The triangle cantilever beam was made from 316L stainless steel, and the density, Young modulus, and Poisson’s ratio were 7850 kg/s, 2 × 10^11^ Pa, and 0.33, respectively. The sensing head was made from nylon material, and the density, Young modulus, and Poisson’s ratio were 1150 kg/s, 2 × 10^9^ Pa, and 0.4, respectively. The input flow velocity was set as 1 m/s, and the output condition was set as 0 Pa. Different simulation conditions lead to different results, so the conditions such as material parameters, input and output direction, input and output velocity, boundary condition, area of flow field, and meshing influenced the simulation results. The simulation results are listed in Table 3. Orthogonal arrays of Taguchi, the signal-to-noise (*S/N*) ratio, and the analysis of variance (ANOVA) were employed to find the optimal levels and to analyze the effect of the cantilever beam structure parameters on probe performance.

**Figure 4:**
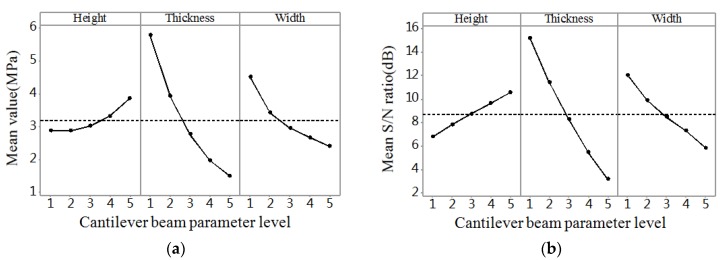
The result graph for stress. (**a**) Mean value; (**b**) Mean *S/N* ratio.

**Figure 5:**
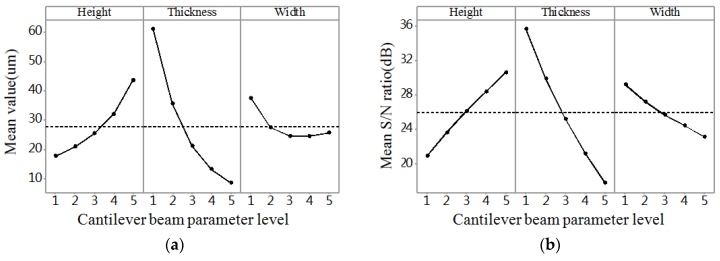
The result graph for deflection. (**a**) Mean value; (**b**) Mean *S/N* ratio.

**Figure 6:**
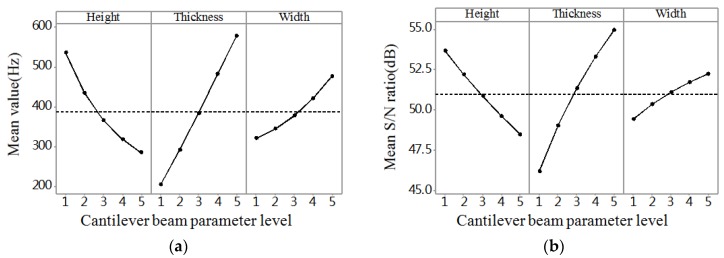
The result graph for frequency. (**a**) Mean value; (**b**) Mean *S/N* ratio.

The range analysis was aimed at illuminating the significant levels of different influencing parameters on the performance of probe. Thus, the most significant parameter could be disclosed according to the results of the range analysis. The range analysis results of stress, deflection, and frequency are shown in Figure 4a, Figure 5a and Figure 6a, respectively. The larger the stress and deflection is, the higher the sensitivity is. Thus, we can see in Figure 4a and Figure 5a that thickness is the most significant parameter influencing sensitivity among the three parameters, and the thinner the thickness is, the higher the sensitivity is. Also, the greater the frequency is, the stronger the robustness is. Thus, we can know from Figure 6a that thickness is the most significant parameter influencing robustness among the three parameters, and the thicker the thickness is, the higher the robustness is.

**Figure 7:**
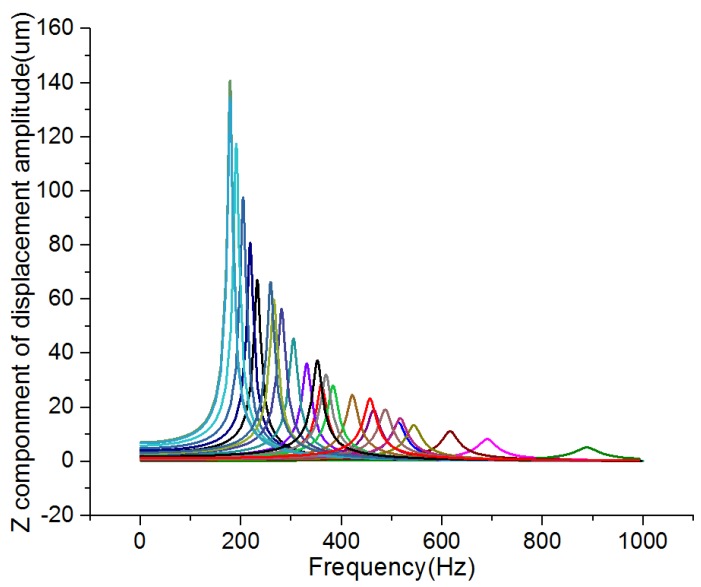
The frequency domain response of 25 combinations.

Taguchi used *S/N* ratio as the quality characteristic of choice. There are three categories of *S*/*N* ratio characteristics when the characteristic is continuous [17,18,19]. First, larger is better:

(10) $$S/N=-10\log\frac{1}{n}(\sum \frac{1}{y^{2}});$$

second, nominal is the best:

(11) $$S/N=10\log\frac{\bar{y}}{s_{y}^{2}};$$

and third, smaller is better:

(12) $$S/N=-10\log\frac{1}{n}(\sum y^{2})$$

where *n* is the number of observations, *y* is the observed data, $\bar{y}$ is the average of observed data, and $s_{y}^{2}$ is the variance of *y*. Large stress and deflection generate high sensitivity, and high natural frequency leads to strong robustness. For all the types of characteristics with the above *S/N* ratio transformation, the higher the *S/N* ratio, the better the result. The *S/N* ratio analysis results of stress, deflection, and frequency are shown in Figure 4b, Figure 5b and Figure 6b, respectively. Similar results can be concluded from the range analysis, that is, the thickness is the most significant parameter influencing sensitivity and robustness among the three parameters, and the thinner the thickness, the higher the sensitivity, and the thicker the thickness, the higher the robustness. Figure 7 shows the frequency domain response of 25 combinations. According to the results from the range analysis and *S/N* ratio analysis, B1C1A5 is the optimal combination of the structure parameters for sensitivity, and B5A1C5 is the optimal combination of the structure parameters for robustness.

The purpose of the ANOVA was to investigate the design parameters that significantly affected the quality characteristic [20]. The total sum of square *SS_T_* from the *S/N* ratio *η* can be calculated as [21]

(13) $$SS_{T}=\sum_{i=1}^{m}{\left(\eta_{i}-\bar{\eta }\right)}^{2}$$

where *m* is the number of the experiment, *η_i_* is the mean *S/N* ratio for the *i*th experiment, and *η* is the mean *S/N* ratio. The sum of squares from the tested parameter *SS_P_* can be calculated as

(14) $$SS_{P}=\sum_{j=1}^{t}\frac{{\left(S\eta_{j}\right)}^{2}}{t}-\frac{1}{m}{\left(\sum_{i=1}^{m}\eta_{i}\right)}^{2}$$

where *P* denotes one of the parameters, *j* is the level number of this parameter *P*, *t* is the repetition of each level of the parameter *P*, and *Sη_j_* is the sum of the *S/N* ratio involving this parameter *P* and level *j*. The sum of square from error *SS_E_* is

(15) $$SS_{E}=SS_{T}-SS_{A}-SS_{B}-SS_{C}.$$

The total degrees of freedom *D_T_* is *D_T_ = m* − 1, and the degree of freedom from tested parameters *D_P_* is *D_P_ = t* – 1. Thus, the degree of freedom from error *D_E_* is *D_E_ = D_T_ − D_A_ − D_B_ − D_C_*. The variance of the tested parameters *V_P_* is *V_P_ = SS_P_/D_P_*, and the variance from the error *V_E_* is *V_E_ = SS_E_/D_E_*. Then, the *F* value for each design parameter is simply the ratio of the mean-of-square deviation to the mean-of-square error:

(16) $$F_{P}=V_{P}/V_{E}.$$

The corrected sum of square *S_P_* can be calculated as

(17) $$S_{P}=SS_{P}-D_{P}V_{E}.$$

Then, the percentage contribution *ρ_P_* can be calculated as

(18) $$\rho_{P}=S_{P}/SS_{T}.$$

Table 4 shows the results of ANOVA for stress, which shows that the thickness parameter is the most significant structure parameter affecting the stress. The width parameter also has a significant effect on stress, and the height parameter has an insignificant effect on stress. The contributions for the stress of the three structure parameters height, thickness, and width are 3.21%, 73.67%, and 16.21%, respectively. Table 5 shows the results of the ANOVA for deflection, which shows that the thickness parameter is the most significant structure parameter affecting the deflection. The height parameter also has a significant effect on deflection, and the width parameter has an insignificant effect on deflection. The contributions for the deflection of the three structure parameters height, thickness, and width are 14.77%, 69.9%, and 2.75%, respectively. Table 6 shows the results of the ANOVA for frequency, which shows that the thickness parameter is the most significant structure parameter affecting the frequency. The height parameter and width parameter also have a significant effect on frequency. The contributions for the frequency of the three structure parameters height, thickness, and width are 26.2%, 58.53%, and 9.54%, respectively.

From the results of the Taguchi method, we conclude that high sensitivity derives from the thin thickness of the cantilever beam and strong robustness derives from the thick thickness of the cantilever beam. This results in a trade-off between sensitivity and robustness. In this paper, we finally chose a height of 20 mm, a thickness of 0.25 mm, and a width of 6 mm in consideration of the comprehensive performance of the designed probe.

## 4. Fabrication and Encapsulation

The fabrication of the designed probe main contained three parts: first, the fabrication of the piezoresistive sensor chip; second, the fabrication of the printed circuit board and signal processing circuit board; and third, the fabrication of the stainless-steel parts, such as the triangle cantilever beam and protective guard.

**Figure 8:**
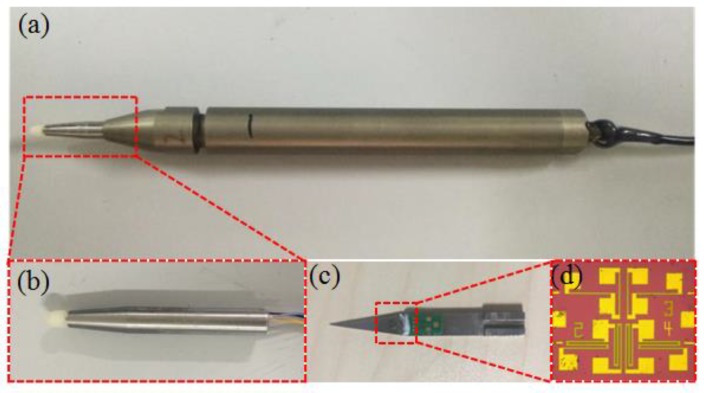
The fabricated probe and MEMS piezoresistive sensor chip. (**a**) The overall of measurement system; (**b**) The fabricated probe; (**c**) The cantilever beam with piezoresistive sensor chip and PCB board on it; (**d**) The picture of piezoresistive sensor chip.

The fabrication of the piezoresistive sensor chip utilized microelectromechanical systems (MEMS) technology. A very thin active layer SOI wafer was used as the starting material. The thickness of the top silicon layer and the buried silicon oxide layer were about 5 μm and 0.4 μm, respectively. The front side of the SOI wafer was implanted by boron ions by means of reactive ion etching (RIE) and the piezoresistors were formed. After that, a high dose of boron ions was diffused and the wafer was annealed. To protect the piezoresistors on the front side of SOI wafer from being corroded, a silicon nitride layer was deposited by low pressure chemical vapor deposition (LPCVD). Then, the metal wire was etched by RIE and contact pads were also formed. The fabricated piezoresistive sensor chip is shown in Figure 8d.

The fabrication of the printed circuit board and signal processing circuit board utilized integrated circuit (IC) technology and microelectronics technology. The fabrication of stainless-steel parts utilized a line-cutting process. To package the piezoresistive sensor chip, some preparatory work had to be completed. First, ultrasonic cleaning of the nylon sensing head, the triangle cantilever beam, the half-cylinder gasket, and the stainless-steel shell was performed using a KH3200DB CNC ultrasonic cleaner. The piezoresistive sensor chip was cleaned using acetone. Then, all of the components were dried on a hot plate. Second, the piezoresistive sensor chip was adhered onto the triangle cantilever beam using M-Bond 610 glue. Also, the printed circuit board was adhered onto the triangle cantilever beam. The fully adhered finished probe is shown in Figure 8c. Third, the gold wire was soldered between the piezoresistive sensor chip contact pad and the printed circuit board contact pad. Then, silica gel was coated on the probe to protect the piezoresistive sensor chip and gold wire. After that, the wire was soldered on the printed circuit board to connect it to the signal processing system. Finally, the triangle cantilever beam and half-cylinder gasket were configured into the stainless-steel shell, and the components were affixed by AB glue. To guarantee that the the probe was leakproof, the shell was filled with silica gel. The packaged probe is shown in Figure 8b. The fabrication and encapsulation of the signal processing system was similar to the fabrication and encapsulation of the probe, and the fabricated probe and signal processing system is shown in Figure 8a.

## 5. Experiments and Results

**Figure 9:**
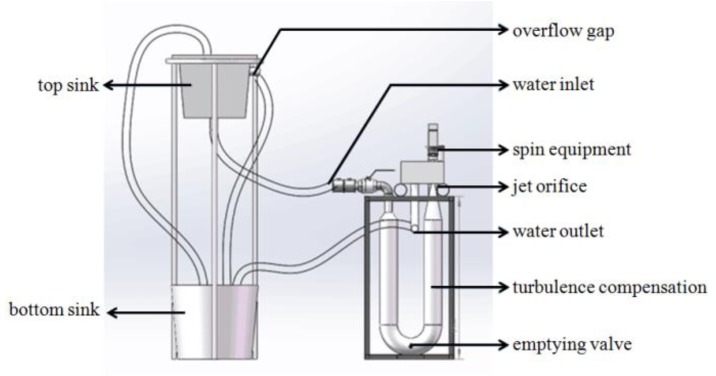
The schematic diagram of flowing cycling experiment system.

**Figure 10:**
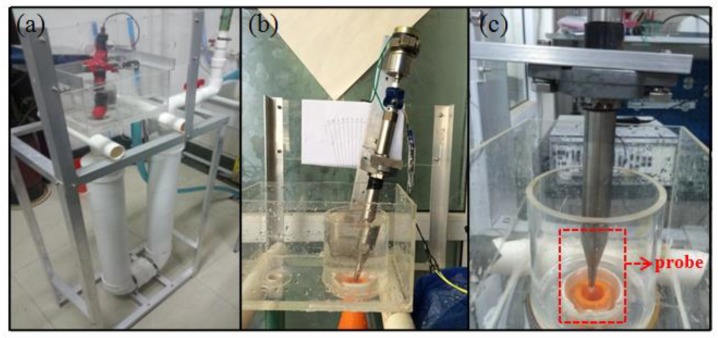
The schematic diagram of experimental setup in the laboratory. (**a**) The flowing cycling experiment system; (**b**) The measurement system in the experiment; (**c**) The designed probe.

To verify the performance of designed probe, tests in the laboratory and in the Bohai Sea were designed and conducted. A flowing cycling experiment system was purposely designed in the laboratory, as shown in Figure 9. The system consisted of top and bottom sinks, an overflow gap, a water inlet and water outlet, spin equipment, a jet orifice, turbulence compensation, and an emptying valve. The probe was installed in the experiment system, and the fluid flow was set under a constant velocity with attack angles from −10° to 10° with a step of 2°. The experimental setup in the laboratory is shown in Figure 10.

**Figure 11:**
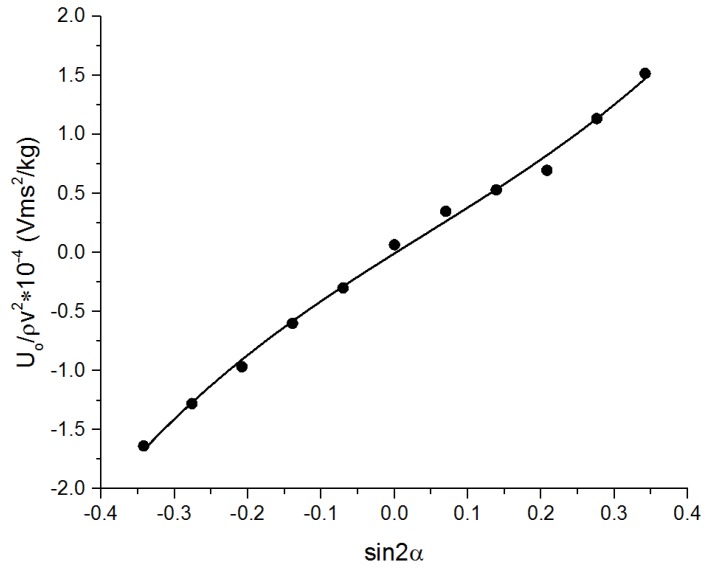
The relationship between the value of *U_o_/ρv^2^* and sin 2*α*.

We obtained 11 sets of data in each experiment, and every set of data includes the angle of attack, the value of sin 2*α*, the output voltage, and the value of *U_o_/ρv*^2^. The sensitivity was calculated using Equation (5), and the experiment results are listed in Table 7. From the experiment results, the relationship between the value of *U_o_/ρv*^2^ and sin2α can be described, which is shown in Figure 11, where the cubic polynomial fitting is used to describe the relationship. The first order coefficient of the polynomial is the sensitivity of probe, which is 3.91 × 10^−^^4^ (Vms^2^)/kg. This sensitivity is larger than that of SPM-38, TJUB, and PNS-03, and close to that of Osborn’s and PNS-06, as listed in Table 1. As a result, we can conclude that the probe designed in this paper can be a probable choice in ocean turbulent kinetic energy dissipation rate measurements.

**Figure 12:**
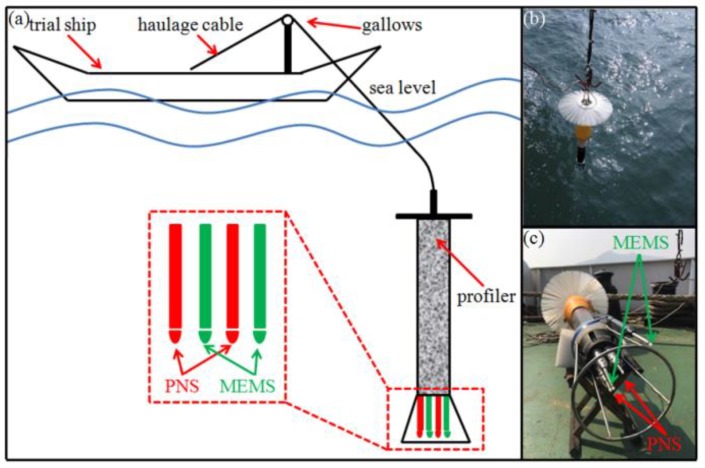
The configuration of the ocean test. MEMS denotes the probe designed in this paper. PNS denotes the PNS series probes. (**a**) The schematic diagram of the ocean test; (**b**) The release of the profiler; (**c**) The configuration of the probes.

**Figure 13:**
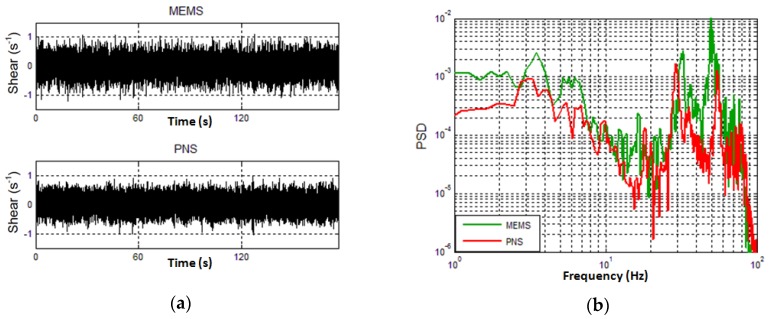
The response results of MEMS and PNS. (**a**) The velocity shear data of MEMS and PNS; (**b**) The power spectrum of velocity shear of MEMS and PNS.

The experiment in the Bohai Sea was designed to verify the practical performance of the designed probe. The probe was carried by an ocean vertical profiler and every profiler carried two probes which were designed in this paper and two PNS-series probes, as shown in Figure 12. A gallows was used for the profiler’s release and recovery. The release velocity was about 1 m/s and the profiler was released at a constant speed. Figure 13a shows the velocity shear data of MEMS and PNS in the experiment, respectively. Note that normalization processing was used to make the comparison between MEMS and PNS. The data collected by MEMS and PNS in the experiment are similar to each other. Figure 13b shows the power spectrum of the velocity shear of MEMS and PNS, respectively. From Figure 13, we can see that the probe designed in this paper has comparable performance with PNS series probes.

**Figure 14:**
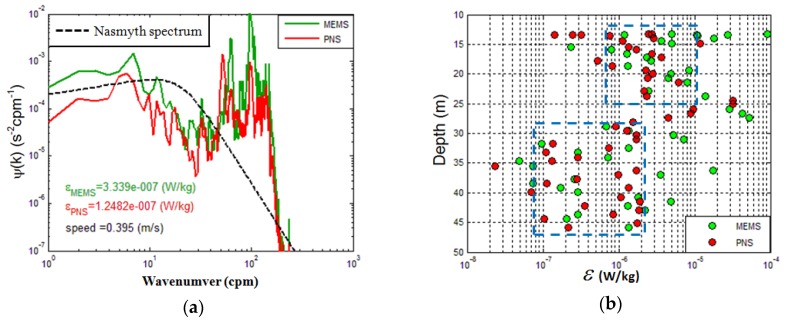
The experiment results of ocean test. (**a**) The power spectrum compared with Nasmyth spectrum. The black dashed curve represents the Nasmyth spectrum; (**b**) The ocean turbulent dissipation rate of MEMS and PNS.

The measured power spectrum is routinely compared to the empirical turbulence spectrum, which was measured by Nasmyth [22], as shown in Figure 14a, and the black dashed curve represents the Nasmyth spectrum. The Nasmyth spectrum is an ideal curve, but in the actual experiments, the results were influenced by noise and vibration, so the experiment results do not match with Nasmyth spectrum strictly. The probes were carried by an ocean vertical profiler, and the release velocity of the profiler and the vibration of the profiler caused by the ship affected the experimental results, so the velocity measurements have spurious contributions from the high-frequency vibrations of towed vehicle. Further, the roll and heave of the ship included large variations of the speeds and depths of the towed vehicles. However, according to Figure 14a, we know that the experiment results both from MEMS and PNS show good agreement with the Nasmyth spectrum. The cut-off frequency is about 100 cpm, and the ocean turbulent kinetic energy dissipation rate of MEMS and PNS are about 3.34 × 10^−7^ W/kg and 1.25 × 10^−7^ W/kg, respectively. The measured ocean turbulent kinetic energy dissipation rates are coincident with the estimated value in this ocean area.

The ocean turbulent kinetic energy dissipation rate was calculated using Equation (9) and the experiment results are shown in Figure 14b. The ocean turbulent kinetic energy dissipation rates measured by MEMS and PNS in the 10–50-m upper mixing area are similar and mainly between 10^−^^8^ W/kg to 10^−^^4^ W/kg. From Figure 14b, we know that the ocean turbulent kinetic energy dissipation rate decreases with the increase of depth. The ocean turbulent kinetic energy dissipation rate is centered on 10^−^^6^–10^−^^5^ W/kg in the 10–25-m upper mixing area and centered on 10^−^^7^–10^−^^6^ W/kg in the 30–50-m upper mixing area, as shown in Figure 14b, marked by the blue dashed box. The experiment results indicate that the probe has similar performance with PNS-series probes once again.

## 6. Conclusions

This paper introduced an ocean turbulent kinetic energy dissipation rate measurement probe. Different from numerous probes that utilize piezoelectric ceramics as the sensing element, the probe designed in this paper utilizes a MEMS piezoresistor as the sensitive element. The structure design and working principle have been introduced, and a signal processing system also been designed and connected with the probe. The Taguchi method has been used to study the influence of the cantilever beam structure parameters on the probe’s performance. Range analysis, signal-to-noise ratio analysis, and analysis of variance were studied. Fluid flowing cycling experiments in the lab revealed that the probe has a sensitivity of 3.91 × 10^−4^ (Vms^2^)/kg. The experiments in the Bohai Sea revealed that the probe has a measurement range between 10^−8^–10^−4^ W/kg. The comparative analysis between the designed probe and the commonly used PNS-series probe shows that the designed probe has equivalent performance with the PNS-series probe. The designed probe can be a strong candidate in marine environmental monitoring.

## Figures and Tables

**Table 1:** The detailed parameters of probes.

Probe	Dimensions (mm)	Withstand Pressure (mm)	Spatial Resolution (mm)	Sensitivity (Vms^2^/kg)
Osborn’s	6.3 in diameter - in length	230	1	4 × 10^−^^4^
SPM-38	9.5 in diameter 127 in length	1000	10	0.57 × 10^−^^4^
TJUB	10 in diameter 63.3 in length	1000	5	2.52 × 10^−^^4^
PNS-03	8 in diameter 77 in length	1000	-	1 × 10^−^^4^
PNS-06	8 in diameter 77 in length	1000	-	4 × 10^−^^4^
This paper	8 in diameter 57 in length	1000	-	3.91 × 10^−^^4^

**Table 2:** Cantilever beam parameters and their levels.

Symbol	Parameter	Unit	Level 1	Level 2	Level 3	Level 4	Level 5
A	Height	mm	16	18	20	22	24
B	Thickness	mm	0.2	0.25	0.3	0.35	0.4
C	Width	mm	4	5	6	7	8

**Table 3:** Experimental layout and results using an L_25_(5^3^) orthogonal array.

Experiment Number	A	B	C	Stress	Deflection	Frequency
1	16	0.2	4	6.86	49.4	232.68
2	16	0.25	5	3.38	20.5	360.31
3	16	0.3	6	2.07	9.93	513.37
4	16	0.35	7	1.27	5.39	690.19
5	16	0.4	8	0.81	3.16	888.59
6	18	0.2	5	5.99	54.3	218.5
7	18	0.25	6	3.23	23.4	331.03
8	18	0.3	7	2	11.7	464.05
9	18	0.35	8	1.25	6.46	616.23
10	18	0.4	4	1.88	8.59	544.03
11	20	0.2	6	5.69	60.4	204.39
12	20	0.25	7	3.13	26.7	304.65
13	20	0.3	8	1.86	13.6	421.86
14	20	0.35	4	2.71	17.2	383.35
15	20	0.4	5	1.71	9.12	516.99
16	22	0.2	7	5.35	67	190.92
17	22	0.25	8	3.08	30.3	281.05
18	22	0.3	4	4.25	35.2	265.61
19	22	0.35	5	2.39	17.8	369.4
20	22	0.4	6	1.53	9.87	487.13
21	24	0.2	8	5.01	74.3	178.4
22	24	0.25	4	6.82	77.1	178.63
23	24	0.3	5	3.62	35.8	259.16
24	24	0.35	6	2.24	18.9	352.26
25	24	0.4	7	1.58	11.6	456.95

**Table 4:** Results of the ANOVA for stress.

Parameter	DOF	Sum of Squares	Variance	*F* Value	*p* Value	Contribution(%)
Height	4	3.432	0.8580	3.79	0.032	3.21
Thickness	4	58.852	14.7131	64.95	0.000	73.67
Width	4	13.654	3.4134	15.07	0.000	16.21
Error	12	2.718	0.2265			6.91
Total	24	78.657				100

**Table 5:** Results of the ANOVA for deflection.

Parameter	DOF	Sum of Squares	Variance	*F* Value	*p* Value	Contribution(%)
Height	4	2108.0	527.00	8.04	0.002	14.77
Thickness	4	8998.2	2249.55	34.32	0.000	69.9
Width	4	605.0	151.26	2.31	0.118	2.75
Error	12	786.7	65.56			12.58
Total	24	12,497.9				100

**Table 6:** Results of the ANOVA for frequency.

Parameter	DOF	Sum of Squares	Variance	*F* Value	*p* Value	Contribution(%)
Height	4	201,236	50,309	28.44	0.000	26.2
Thickness	4	440,802	110,200	62.30	0.000	58.53
Width	4	77,740	19,435	10.99	0.001	9.54
Error	12	21,227	1769			5.73
Total	24	741,004				100

**Table 7:** The experimental results in the flowing cycling experiment system.

*α*	Sin 2*α*	*U_o_*	*U_o_*/*ρv*^2^
−10	−0.342	−1.0261	−1.6367
−8	−0.276	−0.8001	−1.2773
−6	−0.208	−0.6037	−0.9653
−4	−0.139	−0.3727	−0.5982
−2	−0.070	−0.1842	−0.2984
0	0.000	0.0457	0.0669
2	0.070	0.2241	0.3504
4	0.139	0.3388	0.5328
6	0.208	0.4427	0.6980
8	0.276	0.7183	1.1359
10	0.342	0.9579	1.5186
